# *EGFR*-Mutant Lung Adenocarcinoma Cell-Derived Exosomal miR-651-5p Induces CD8+ T Cell Apoptosis via Downregulating BCL2 Expression

**DOI:** 10.3390/biomedicines13020482

**Published:** 2025-02-15

**Authors:** Chao Zhao, Lei Cheng, Aiwu Li, Haowei Wang, Xuefei Li, Jun Xu

**Affiliations:** 1 Department of Lung Cancer and Immunology, Shanghai Pulmonary Hospital, School of Medicine, Tongji University, Shanghai 200433, China; dlyzcr-zc@163.com (C.Z.); chenglei_2008@126.com (L.C.); 2 Department of Medical Oncology, Shanghai Pulmonary Hospital, School of Medicine, Tongji University, Shanghai 200433, China; 3 Shanghai East Hospital, School of Medicine, Tongji University, Shanghai 200120, China

**Keywords:** non-small cell lung cancer, epidermal growth factor receptor, exosomes, apoptosis

## Abstract

**Background**: The efficacy of programmed cell death 1 (PD-1) or ligand 1 (PD-L1) inhibitors in epidermal growth factor receptor (*EGFR*)-mutant non-small cell lung cancer (NSCLC) patients is not satisfactory. Studies have indicated that the ratio of CD8+ tumor infiltration lymphocytes (TILs) was associated with immunotherapy efficacy; however, it was significantly lower in *EGFR*-mutant than wild type patients. The underlying mechanisms need to be studied. **Methods**: Database analysis, clinical specimens, small RNA sequencing, and single-cell sequencing were used to analyze miRNA expression and immune cell infiltration. Cell co-culture and flow cytometry were conducted to detect immune cell apoptosis. The mouse model was performed to analyze the influence of miR-651-5p antagomirs on the tumor microenvironment. **Results**: The miR-651-5p was found to be highly expressed in *EGFR*-mutant lung adenocarcinoma cell-derived exosomes, which could promote CD8+ T cell apoptosis, while the miR-651-5p inhibitor decreased the ratio of PC9-secreted exosomes and induced apoptosis. Mechanistically, the EGFR signaling pathway promoted the expression of miR-651-5p by activating the transcription factor Fos proto-oncogene (FOS) in *EGFR*-mutant lung adenocarcinoma cell lines. B-cell lymphoma 2 (BCL2) was the target of miR-651-5p, and miR-651-5p could promote T cell apoptosis by inhibiting BCL2 expression. In addition, the miR-651-5p antagomir increased T cell infiltration and enhanced the efficacy of the PD-1 inhibitor treating the *EGFR*-mutant lung adenocarcinoma humanized mouse model. **Conclusions**: *EGFR*-mutant lung adenocarcinoma promotes T cell apoptosis through exosomal miR-651-5p. miR-651-5p antagonists increase immune cell infiltration and enhance the anti-tumor effect of PD-1 inhibitor, suggesting a new combination therapy to improve the efficacy of immunotherapy in *EGFR*-mutant NSCLC patients.

## 1. Introduction

Lung cancer is the leading cause of tumor-related death worldwide, and non-small cell lung cancer accounts for 80–85% [1]. Epidermal growth factor receptor (EGFR) tyrosine-kinase inhibitors (TKIs) are the standard first-line treatment for *EGFR*-mutant non-small cell lung cancer (NSCLC) patients; however, drug resistance eventually appears, such as gefitinib, afatinib, and osimertinib, which limits the survival of these patients [2]. Immunotherapy based on programmed cell death 1/ligand 1 (PD-1/PD-L1) inhibitors prolongs the survival of *EGFR* wild type NSCLC patients; however, the efficacy and prognosis in *EGFR*-mutant NSCLC patients are not satisfactory [3,4,5,6]. The underlying mechanisms still need to be explored. Studies showed that the tumor microenvironment (TME) played important roles on immunotherapy response. To distinguish the features between *EGFR*-mutant and wild type NSCLC could be an effective strategy to reveal the mechanism.

*EGFR*-mutant NSCLC exhibited distinctive TME features in PD-L1 expression, tumor mutation burden (TMB), and CD8+ tumor infiltration lymphocytes (TILs) compared with wild type NSCLC [7]. CD8+ TILs, as an important component in the TME, are critical cells for PD-1/PD-L1 inhibitors to exert their effects in immunotherapy. The abundance was associated with good immunotherapy effects [8] and could be a better parameter than PD-L1 expression and TMB to predict immunotherapy effects [9]. However, the ratio of CD8+ TILs was significantly lower in *EGFR*-mutant than wild type patients [5,10,11]. To increase the ratio of CD8+ TILs is thought be a potential strategy to improve the efficacy of PD-1/PD-L1 inhibitors.

T cells undergo apoptosis in TMEs, which may impede the infiltration number and anti-tumor activity. To inhibit the apoptosis of TILs could strengthen immune checkpoint inhibitor (ICI) efficacy [12,13,14,15]. Exosomes are widely known extracellular vesicles, which can regulate immune reactions in the TME as well as induce T cell apoptosis [16,17]. miRNAs, as important components in exosomes, are also found to regulate immune cell functions including promoting apoptosis in NSCLC and other tumors [18,19,20,21]. Our former study showed that *EGFR*-mutant NSCLC exosomes had a stronger ability of promoting CD8+ T cell apoptosis than wild type cell-derived exosomes [11]; however, the underlying mechanism was unknown.

In this study, we investigated the differently expressed miRNAs between *EGFR*-mutant and wild type NSCLC exosomes, explored mechanisms of the miRNA expression, regulation, and promotion of apoptosis, and also studied the anti-tumor function of the miRNA inhibitor in humanized mouse models. This study is in some degree to reveal the mechanisms of poor efficacy of ICIs treating *EGFR*-mutant NSCLC and to provide some indications for immunotherapy in this group.

## 2. Materials and Methods

### 2.1. Patients

The peripheral blood of thirty-five NSCLC patients before treating with a PD-1 inhibitor single drug was collected from 2016 to 2018. Plasma and blood cells were separated by centrifuge and stored at −80 °C. This study was approved by the Ethics Committee of Shanghai Pulmonary Hospital (K20-288).

### 2.2. Peripheral Blood Monocytes Extraction

Peripheral blood of healthy volunteers was collected, and peripheral blood monocytes (PBMCs) were separated by Ficoll-Paque PREMIUM. Briefly, fresh blood was diluted by PBS. A Ficoll reagent was added at the bottom of centrifuge tube, and the diluted blood was added carefully on the top of the reagent. After centrifuge, the mononuclear cell at the interface was collected in a new tube. PBS was added, fully mixed, and then centrifuged. The precipitate was resuspended by PBS and centrifuged. The precipitate was resuspended directly for use. The main reagents in this study are shown in Supplementary Table S1.

### 2.3. Tumor Cell and PBMC Co-Culture

PBMCs were placed in a 12-well plate and cultured by an RPMI 1640 medium supplemented with 10% fetal bovine serum, 100 U/mL of penicillin, and 100 mg/mL of streptomycin for 4–6 h. Tumor cells were cultured by DMEM supplemented with 10% fetal bovine serum, 100 U/mL of penicillin, and 100 mg/mL of streptomycin. Tumor cells of 5 × 10^4^ in 1 mL and PBMCs of 5 × 10^5^ tumor cells in 100 µL were co-cultured in a 12-well plate with the number of tumor cells to PBMCs at 1:10 for 24 h. The cells were collected in a 1.5 mL tube and used to conduct the flow cytometry assay or tumor killing assay.

### 2.4. CD8+T Cell Separation

CD8 microbeads were used to separate CD8+ T cells from PBMCs. Briefly, PBMCs about 1 × 10^7^ cells were resuspended in an 80 μL buffer. CD8 microbeads of 20 μL were added to the PBMCs, mixed well, and incubated for 15 min at 4 °C. Then, the mixture was washed and centrifuged at 300× *g* for 10 min. The precipitate was resuspended and separated by MACS columns to obtain CD8+ T cells. CD8+ T cells were resuspended in an RPMI 1640 medium and cultured in a 12-well plate after cell counting.

### 2.5. Apoptosis Detection

The cultured cells were harvested by centrifuge at 400× *g* for 5 min in a 1.5 mL tube. The precipitate was resuspended by Dulbecco’s PBS (DPBS) and centrifuged at 400× *g* for 5 min. Then, the precipitate was stained by fixable viability stain 620 at 4 °C for 30 min. After centrifuge, the cells were resuspended by stain buffer and added Fc block. After centrifuge, the precipitate was resuspended by an apoptosis stain buffer and stained by antibodies (Supplementary Table S1) at 4 °C for 30 min. Finally, the mixture was centrifuged, resuspended by stain buffer, and analyzed by flow cytometry.

### 2.6. Cell Viability Detection

Cells were placed in a 96-well plate, and cultured for 24 h. Then, the supernatant was discarded, and the 100 μL fresh culture medium containing different doses of drugs was added. After 24 h, the supernatant was discarded, and a 90 μL fresh culture medium and a 10 μL cell counting kit-8 reagent were added to each well and detected by a microplate reader.

### 2.7. Exosome Extraction and Identification

Exosomes were extracted from cell culture mediums as previously described [11]. The plasma exosome was extracted by a plasma exosome extraction kit. Briefly, add 4 μL of reagent C into 200 μL of plasma, mix well, and incubate at 37 °C for 15 min. After being centrifuged at 10,000× *g* for 10 min, the supernatant was transferred to a new tube. Add 50 μL of reagent A to the sample, and incubate it at 4 °C for 30 min. After centrifuge, the pellet was resuspended by PBS, added 50 μL reagent B, and incubated at 4 °C for 30 min. Finally, the sample was centrifuged at 3000× *g* for 10 min, and the pellet was resuspended by PBS and stored at −80 °C. The exosomes were pictured by transmission electron microscope and conducted particle size analysis and protein marker (TSG101 and HSP70) detection to identify the quality of exosomes.

### 2.8. Exosome Uptake by PBMCs

About 5 × 10^5^ PBMCs in 1 mL were cultured in a 12-well plate. Exosomes of 10 μg were labeled by ESQ-R-001 and then used to treat PBMCs for 48 h. The PBMCs were harvested and washed twice by PBS and then stained by DAPI. After washing, the PBMCs were resuspended and pictured using a fluorescence microscope (Echo Revolve, San Diego, CA, USA).

### 2.9. T Cell-Mediated Cancer Cell Killing Assay

PBMCs, exosomes, or miR-651-5p inhibitor/mimic-transfected PBMCs were co-cultured with tumor cells to detect the tumor-killing effect. The procedure was conducted as previously described using the xCELLigence RTCA instrument (ACEA Biosciences, San Diego, CA, USA) [21].

### 2.10. miRNA Extraction and qRT-PCR

Cell and exosome miRNAs were extracted using the miRNeasy Mini Kit. Briefly, QIAzol was added to the samples and incubated at room temperature. Then, chloroform was added, fully mixed, and centrifuged. An appropriate volume of 100% ethanol was added to the upper aqueous phase in a new tube, transferred to the column, and centrifuged. After being washed by the buffers, miRNAs were collected in an RNase-free tube. A Bulge-Loop miRNA qRT-PCR Starter Kit was used to conduct miRNA reverse transcription and PCR reaction. miRNA RT primers and PCR primers were commercial reagents. The PCR reactions were conducted on Agilent 3000P (Agilent, Santa Clara, CA, USA).

### 2.11. Small RNA Sequencing

Small RNA sequencing was conducted in NSCLC cell line exosomes to identify the differential expression miRNAs (Novogene, Beijing, China). Briefly, RNA quantity and purity were accessed. Then, a Small RNA Sample Pre Kit was used to construct the library. After quality detection, the library was sequenced, and the differential expression miRNAs were analyzed.

### 2.12. Public Database Analysis

Public databases were used in this study. The TCGA database (https://portal.gdc.cancer.gov/, accessed on 19 November 2022) was used to analyze miRNA expression in *EGFR* mutation and wild type lung adenocarcinoma tumor tissues, as well as in different cancers. Transcription factors were predicted in the PROMO (http://alggen.lsi.upc.es/cgi-bin/promo_v3/promo/promoinit.cgi?dirDB=TF_8.3, accessed on 10 May 2021) and Genecard website (https://www.genecards.org/, accessed on 10 May 2021). The Fos proto-oncogene (FOS) binding site was predicted in the JASPAR website (https://jaspar.elixir.no/, accessed on 11 October 2021). The potential targets and binding sites on the mRNAs were predicted in the TARGETSCAN website (https://www.targetscan.org/vert_80/, accessed on 10 May 2021). The ENCORI database (https://rnasysu.com/encori/, accessed on 2 June 2023) was used to exhibit miR-651-5p expression in different tumor and normal tissues. The Targetscan website was used to predict potential miR-651-5p binding genes.

### 2.13. Cell Transfection

miRNA inhibitor/mimic transfection and siRNA transfection were conducted by a riboFECT CP Transfection Kit. The plasmid transfection was conducted by LipoFiter3.0. After transfection for 48 h, cells were collected for the research below.

### 2.14. mRNA Extraction and qRT-PCR

Total RNAs were extracted by RNAiso Plus and reversed to cDNA by RevertAid First Strand cDNA. PCR primers are listed in Supplementary Table S2. The PCR reactions were conducted on Agilent 3000P.

### 2.15. Luciferase Report Assay

The 293T cells about 2 × 10^3^ in 100 µL were counted and cultured in a 96-well plate. The plasmid, culture medium, and transfection regent were mixed and incubated at room temperature for 20 min. Half-volume of the cell culture medium was discarded, and an equal volume of the mixture was added to the well. After 48 h, the firefly luminescence and renilla luminescence were detected.

### 2.16. Animal Model

Humanized female huHSC-NOG-EXL mice were purchased from Beijing Charles river Co., Ltd. (Beijing, China), and kept in Shanghai Pulmonary Hospital Animal House. PC9 cells of 100 µL containing 1 × 10^6^ cells were inoculated subcutaneously into the right flank of mice. Tumor long axis and short axis were measured twice every week. Tumor volume was calculated using (long axis) × (short axis)^2^/2. hsa-miR-651-5p antagomirs (Antagomir group) or the negative controls (Control group) of 10 nM were intratumorally injected twice every week. The PD-1 inhibitor pembrolizumab of 200 µg/mouse was intraperitoneally injected twice every week (PD-1 antibody group). The PD-1 antibody and Antagomir group was treated by pembrolizumab and hsa-miR-651-5p antagomirs as mentioned above. After three weeks, the mice were sacrificed, and the tumors were harvested to conduct paraffin embedding or sing-cell sequencing.

### 2.17. Single-Cell Sequencing

The fresh tumor tissue was stored in the GEXSCOPE^®^ Tissue Preservation Solution (Singleron Biotechnologies Co., Ltd., Nanjing, China) and transported to the Singleron lab (Singleron Biotechnologies Co., Ltd., Nanjing, China) on ice as soon as possible. The specimens were minced into 1–2 mm pieces and then digested. After digestion, using 40-micron sterile strainers to filter the samples and centrifuging the samples to obtain the sediment, the sediment was resuspended in PBS and red blood cell lysis buffer was added to remove the red blood cells. The solution was then centrifuged and resuspended in PBS. The sample was stained with trypan blue and microscopically evaluated. Single-cell suspensions were prepared, and single-cell RNA sequencing (scRNA-seq) libraries were constructed by the GEXSCOPE^®^ Single-Cell RNA Library Kit (Singleron Biotechnologies Co., Ltd., Nanjing, China). The sequencing was conducted on Illumina HiSeq X. Immune cell subtype analysis and gene expressions were also performed by the platform of Singleron Biotechnologies.

### 2.18. Immunohistochemistry Staining

The mice tumor tissues were embedded to wax blocks and then were cut into 5 μm slices. CD8 and cleaved-caspase-3 immunohistochemistry (IHC) antibodies were used to detect CD8+ TILs and the apoptosis of the tumor cells and immune cells.

### 2.19. Statistical Analysis

Flow cytometry data analyses were performed using GraphPad Prism7.0 (San Diego, CA, USA). Paired Student’s *t* test was used to analyze the ratio of apoptosis between different groups. Comparisons of miRNA expression in different clinical pathological characteristics were evaluated by Pearson Chi-square test or Fisher’s exact test. The Kaplan–Meier survival probability was drawn, and a log-rank test was used to calculate the significant differences. IHC stain analysis was performed using an unpaired Student’s *t* test or Fisher’s exact test. The two-sided significance level was set at *p* < 0.05.

## 3. Results

### 3.1. NSCLC Cell Lines Induced Immune Cell Apoptosis

**Figure 1:**
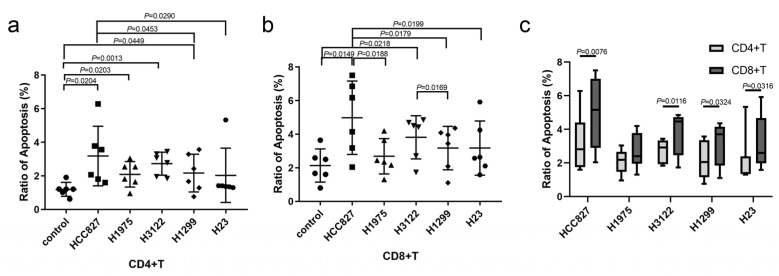
NSCLC cell lines induced immune cell apoptosis. *EGFR*-mutant (HCC827 and H1975) and wild type NSCLC cell lines (H3122, H1299, and H23) co-cultured with PBMCs for 24 h, and, then, the apoptosis of immune cells was detected. The apoptosis ratio of CD4+ T cells (**a**) and CD8+ T cells (**b**) between different cell lines, and the comparison in the same cell line (**c**). The significant differences were shown in the figure.

To determine the effect of lung cancer on immune cell apoptosis, NSCLC cell lines were co-cultured with PBMCs from healthy volunteers. We observed that tumor cells could induce T cell apoptosis (Figure 1). The promoting apoptosis ability of *EGFR* mutation cell line HCC827 (harboring EGFR exon 19 deletion) was stronger than wild type cell line H1299 and H23 (Figure 1a,b). The ability of HCC827 was also stronger than H1975 (harboring EGFR L858R and T790M mutation) for CD8+ T cells but not CD4+ T cells. The ability was not significantly different between *EGFR* wild type cell lines, except that it was stronger in H3122 (anaplastic lymphoma kinase *ALK* rearrangement cell line) than H1299 for CD8+ T cells but not CD4+ T cells. Moreover, the ratios of apoptosis were stronger in CD8+ T cells than in CD4+ T cells (Figure 1c). These findings suggest that NSCLC promotes CD8+ T cell apoptosis, and the ability was stronger in *EGFR*-mutant NSCLC than wild type NSCLC.

### 3.2. Tumor Cell-Derived Exosomes Induced T Cell Apoptosis

**Figure 2:**
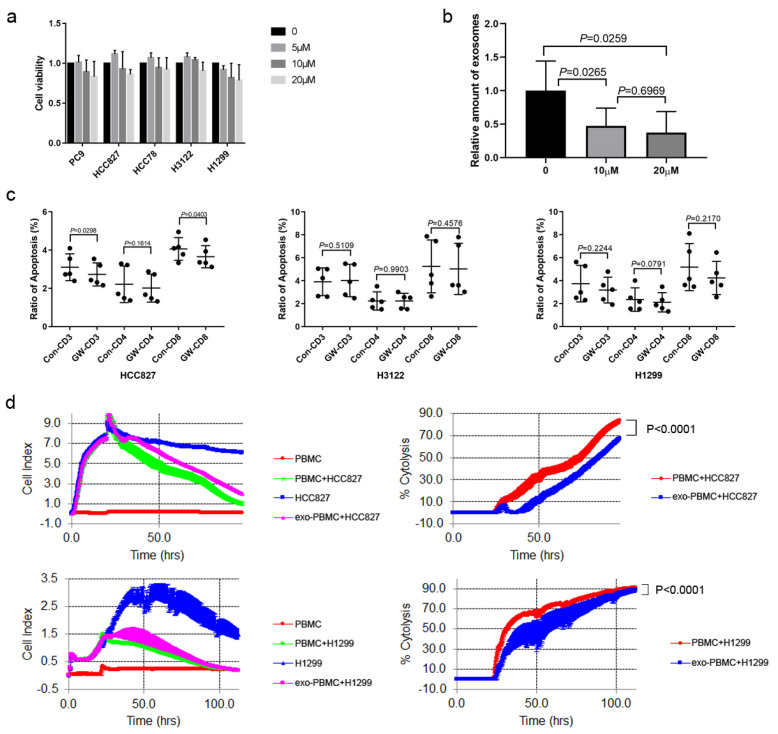
Exosomes induced T cell apoptosis: (**a**) Cell viability is detected after different doses of GW4869 treating NSCLC cell lines. (**b**) Different doses of GW4869 are used to treat HCC827 cells, and the generation of exosomes are down-regulated. (**c**) NSCLC cells are treated by 10 μM of GW4869, and the ability of exosomes promoting T cell apoptosis is detected. (**d**) PBMCs or HCC827/H1299-derived exosomes treat PBMCs co-cultured with HCC827/H1299 cells, respectively, and the cell proliferation (cell index)/tumor-killing effect (cytolysis) are detected by the RTCA method. The tumor killing effect is decreased when co-cultured with exosome-treated PBMCs compared with those not treated. Exo-PBMC, PBMC treated with exosomes from HCC827 or H1299.

To explore whether exosomes could influence immune cell apoptosis, GW4869 was used to inhibit exosome generation. The results showed that it had a slight influence on cell proliferation, with no significant differences (Figure 2a). Exosomes were extracted from cell culture mediums and identified (Supplementary Figure S1a–c). Exosomes were labeled to treat PBMCs, and they could be absorbed (Supplementary Figure S1d). GW4869 of 10 μM could decrease exosome generation about 50%, which had a similar effect with 20 μM (Figure 2b). We used 10 μM to treat NSCLC cells in the following study. It was found that GW4869 down-regulated the ability of promoting apoptosis of HCC827 cell exosomes. The effect was significant on CD8+ T cells but not CD4+ T cells. In other cell lines, GW4869 had no significant influences (Figure 2c). We also detected whether exosomes could influence T cell anti-tumor activity and found that the cytotoxicity was decreased when treated by HCC827 or H1299 exosomes compared with those not treated, indicating that exosomes impaired the T cell tumor-killing function (Figure 2d). The damage to cytotoxicity was maintained at about 20% during the 72 h for HCC827 exosomes, while the damage to the cytotoxicity gradually decreased for H1299 exosomes. These results indicate that tumor exosomes could induce CD8+ T cell apoptosis and impair the anti-tumor function.

### 3.3. miR-651-5p Was Associated with CD8+ T Cell Apoptosis

**Figure 3:**
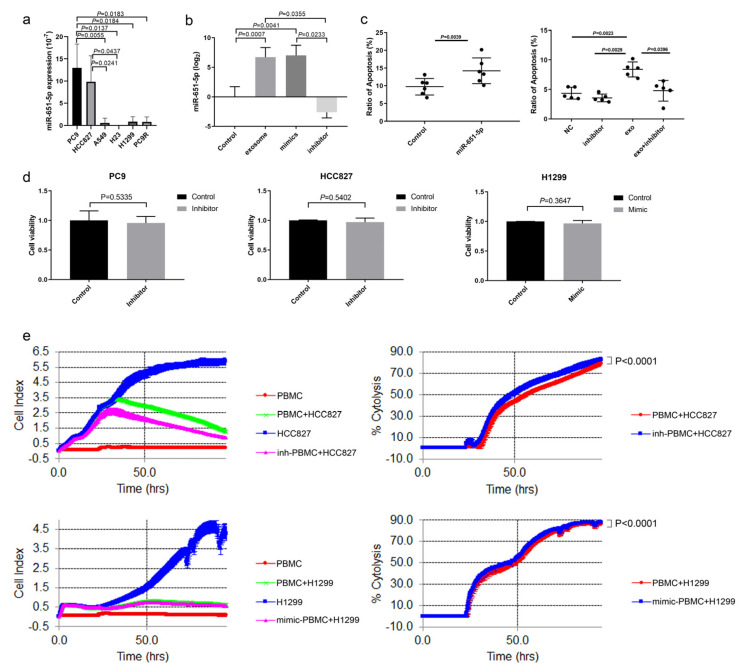
miR-651-5p promoted CD8+ T cell apoptosis: (**a**) miR-651-5p expression is detected in cell line-derived exosomes by qRT-PCR. (**b**) PC9 cell exosomes, miR-651-5p mimics, and inhibitors are used to treat/transfect CD8+T cells, and the expression of miR-651-5p is detected. (**c**) The transfection of miR-651-5p mimics to CD8+ T cells increase the ratio of apoptosis; the exosomes increase the ratio of CD8+ T cell apoptosis, and the addition of the inhibitor decreases promoting the apoptosis function of exosomes. (**d**) PC9 and HCC827 are transfected by the miR-651-5p inhibitor, and H1299 is transfected by miR-651-5p mimics for 24 h. Then, cells are digested and placed on a 96-well plate for 24 h, followed by cell proliferation detection. (**e**) miR-651-5p mimics or inhibitors are transfected to PBMCs, PBMCs are co-cultured with tumors, and cell proliferation and tumor-killing cytotoxicity are detected. Inh-PBMC, PBMC transfected with miR-651-5p inhibitors; and mimic-PBMC, PBMC transfected with miR-651-5p mimics.

miRNAs, as important components in exosomes, play significant roles in immune regulation. To explore whether and which exosomal miRNAs promoted immune cell apoptosis, small RNA sequencing was performed. The results showed that the expression of miR-651-5p was higher in *EGFR*-mutant than wild type NSCLC cell line exosomes (Supplementary Figure S2). Subsequently, the result was confirmed by qRT-PCR in exosomes (Figure 3a) and cell lines (Supplementary Figure S3a). TCGA database analysis showed that miR-651-5p was significantly higher in *EGFR*-mutant than wild type lung adenocarcinoma tissues (Supplementary Figure S3b). The expression of miR-651-5p was analyzed in different tumors, and it was shown that the expression was significantly higher in most cancers than in the normal tissues (Supplementary Table S3). It was also higher in lung adenocarcinoma (LUAD) and lung squamous carcinoma (LUSC) than the normal tissues (Supplementary Figure S3c). We also detected the expression in 35 treatment naïve NSCLC biopsy tissues and found that it was higher in *EGFR*-mutant than wild type tissues (*p* = 0.0237) (Supplementary Figure S3d). Pathway analysis showed that miR-651-5p was closely associated with apoptosis (Supplementary Table S4). miR-651-5p mimic and PC9 (harboring *EGFR* exon 19 deletion) exosomes promoted CD8+ T cell apoptosis, while the miR-651-5p inhibitor decreased the ratio of PC9 exosome-induced apoptosis (Figure 3b,c). miR-651-5p mimics and inhibitors had no significant influence on the proliferation of H1299, PC9 or HCC827, respectively (Figure 3d). Further, we explored whether miR-651-5p could affect T cell anti-tumor function and found that the inhibitor increased the anti-tumor cytotoxicity function of T cells in HCC827 cells. However, the mimics also slightly increased the anti-tumor function in H1299 cells, indicating that miR-651-5p may not have significant influence on the tumor-killing function, or it may have different influences on different tumors (Figure 3e). These findings suggest that *EGFR*-mutant NSCLC promotes T cell apoptosis through exosomal miR-651-5p, but the effect on T cell anti-tumor function is not significant.

### 3.4. miR-651-5p Expression Was Regulated by EGFR and FOS

**Figure 4:**
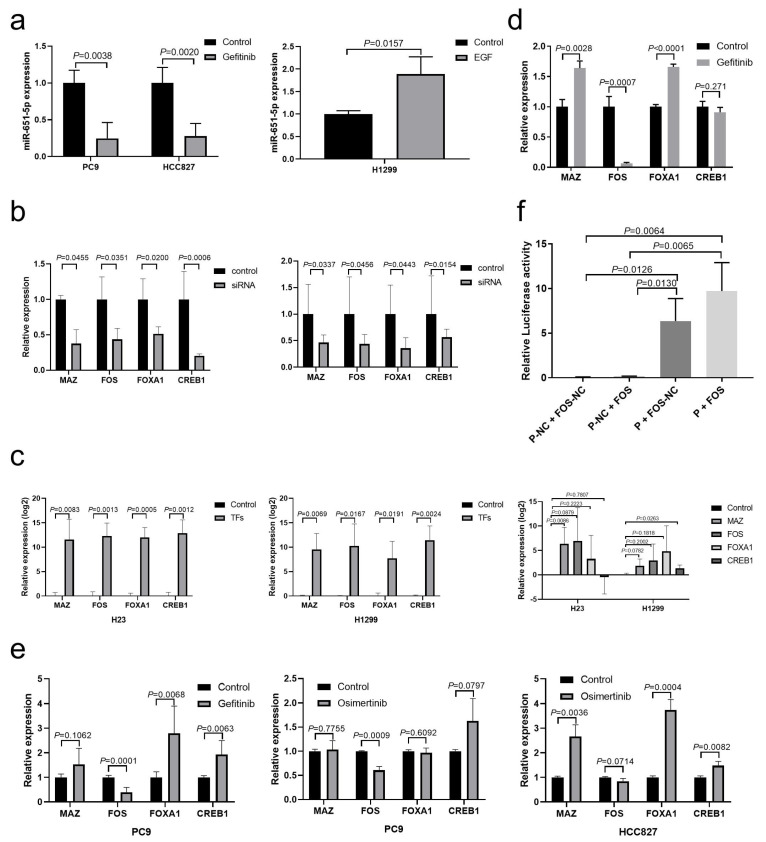
The regulation of miR-651-5p expression: (**a**) Gefitinib and EGF are used to treat PC9/HCC827 and H1299 for 24 h, respectively, and the expression of miR-651-5p is decreased and increased accordingly. (**b**) siRNAs of the potential TFs are transfected to the PC9 cell, and the interference efficiencies (**left**) and miR-651-5p expression (**right**) are detected. (**c**) The over-expression plasmids of the TFs are transfected to H23 (**left**) and H1299 cells (**middle**), the over-expression efficiencies and miR-651-5p expression are detected (**right**). (**d**) HCC827 is treated by 10 nM of Gefitinib for 24 h, and RNA sequencing is conducted. The expression of the four TFs is exhibited. (**e**) PC9 and HCC827 cells are treated by Gefitinib and Osimertinib for 24 h, and the expression of TFs is detected. (**f**) FOS overexpression (FOS) or the control plasmid (FOS-NC) are transfected to 293T cells, and the miR-651-5p upstream sequence plasmid (P) or control (P-NC) is also used to transfect the cells. Then, fluorescence activity is detected. FOS up-regulated the fluorescence activity of the miR-651-5p upstream sequence plasmid compared with FOS-NC.

Next, we explored the regulation mechanism of miR-651-5p in *EGFR*-mutant NSCLC. Since miR-651-5p expression was higher in *EGFR*-mutant cell lines than wild type cell lines, the EGFR-TKI and epidermal growth factor (EGF) were used to treat NSCLC cell lines to explore whether it was regulated by the EGFR signaling pathway (Figure 4a). Gefitinib is a small molecular compound that targets mutant EGFR and inhibits the EGFR pathway. Here, it decreased the miR-651-5p expression in *EGFR*-mutant cell lines, and EGF increased the expression in H1299 cells, indicating that the EGFR pathway could regulate miR-651-5p expression. Then, to investigate which transcription factors (TFs) could regulate miR-651-5p expression, we searched PROMO and Genecard databases and found four potential TFs (Supplementary Figure S4). The expression of TFs was knockdown in PC9 cell, and the expression of miR-651-5p decreased accordingly (Figure 4b). In H23 and H1299 cells, miR-651-5p expression increased when the TFs were over-expressed (Figure 4c). To further study which TFs played important roles in *EGFR*-mutant cells, RNA sequencing was conducted in HCC827 treated by Gefitinib, and the result showed that only FOS was down-regulated significantly (Figure 4d). Gefitinib and Osimertinib were used to treat PC9 and HCC827 cells to validate the result, showing that only FOS was down-regulated (Figure 4e). In order to validate whether FOS could bind the miR-651-5p upstream sequence, the binding site was predicted (Supplementary Table S5), and the plasmid was constructed on a GV238 plasmid (Supplementary Figure S5a). The luciferase report assay showed that FOS could strengthen the transcription activity, indicating FOS could enhance miR-651-5p expression (Figure 4f). Taken together, these results indicate that the EGFR signaling pathway promotes the expression of miR-651-5p by activating the transcription factor FOS in EGFR-mutant NSCLC.

### 3.5. miR-651-5p Could Target BCL2

**Figure 5:**
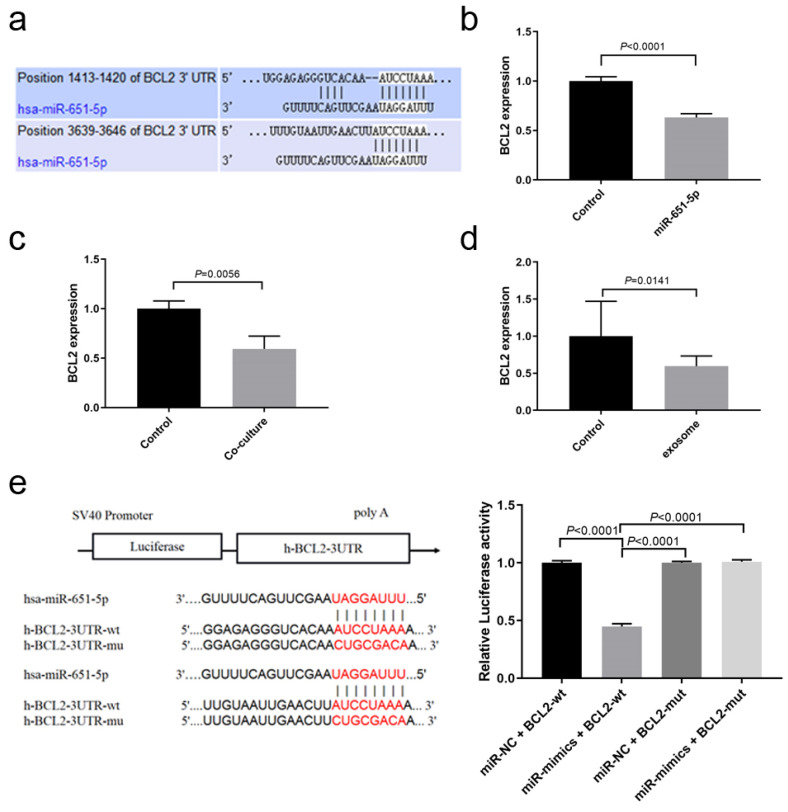
miR-651-5p could target BCL2: (**a**) The Targetscan website predicts that miR-651-5p could target BCL2 3′UTR. miR-651-5p mimics (**b**), PC9 cell co-cultured with CD8+ T cells (**c**), or PC9 cell exosomes (**d**) are used to treat CD8+ T cells for 24 h, and, then, the expression of BCL2 is detected. The treatment down-regulates the expression of BCL2. (**e**) The wild type and mutant BCL2-3′UTR plasmids are constructed (left). The plasmids and miR-651-5p mimics or negative control are transmitted to 293T cells. The miR-651-5p mimics decrease the wild type plasmid luciferase activity, while it did not decrease the mutant plasmid luciferase activity. miR-NC, miR-651-5p negative control; miR-mimics, miR-651-5p mimics; BCL2-wt, BCL2 3′UTR wild type sequence plasmid; and BCL2-mut, BCL2 3′UTR mutant sequence plasmid.

To further clarify how miR-651-5p regulates T cell apoptosis, miR-651-5p targets were predicted in the Targetscan website, and it was found that B-cell lymphoma 2 (BCL2) was one of the targets related to apoptosis pathways (Figure 5a). Using the miR-651-5p mimic or PC9 exosomes to treat CD8+ T cells, or the PC9 cell line co-culture with CD8+ T cells, the BCL2 expression in CD8+ T cells was down-regulated (Figure 5b–d). We constructed wild type and mutation plasmids of miR-651-5p potential binding sites on BCL2 3′UTR on the pSI-check2 plasmid (Supplementary Figure S5b) and detected the influence of miR-651-5p on them. The results showed that the miR-651-5p mimic could down-regulate wild type plasmid luciferase activity, while it did not influence the mutant plasmid luciferase activity, suggesting that miR-651-5p could target BCL2 (Figure 5e). Therefore, miR-651-5p may promote T cell apoptosis by inhibiting BCL2 expression.

### 3.6. miR-651-5p Antagomir Potentiated PD-1 Inhibitor Anti-Tumor Effect

**Figure 6:**
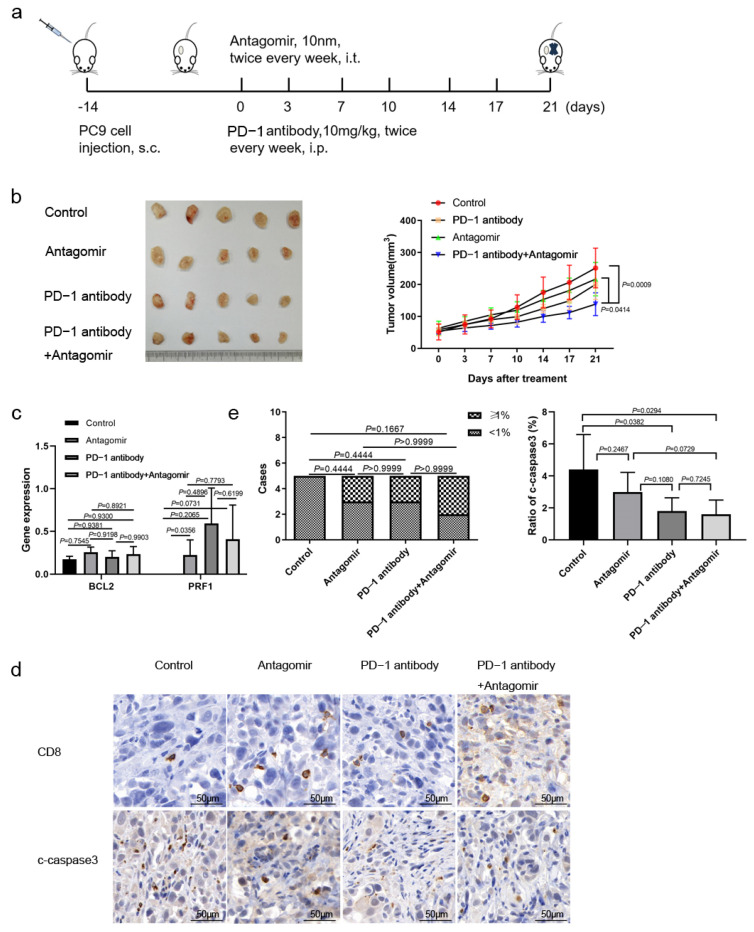
The anti-tumor effect of miR-651-5p in the humanized mouse model: (**a**) The diagram of the mouse model. (**b**) After treatment for 21 days, five tumors of each group are harvested, and the variations in tumor volumes are calculated and analyzed. The combination treatment significantly inhibited tumor growth than the control as well as the antagomir group. (**c**) The expression of the apoptosis-associated gene BCL2 and the cytotoxicity-associated gene PRF1 in T cells between different groups are detected by single-cell sequencing. CD8 and cleaved-caspase 3 (c-caspsse3) are stained by IHC (**d**), and ratios in immune cells are calculated (**e**). CD8+ T cell infiltration is divided into two categories: <1% and ≥1% (**left**). The ratio of cleaved-caspase3 (c-caspase3) is analyzed (**right**).

**Figure 7:**
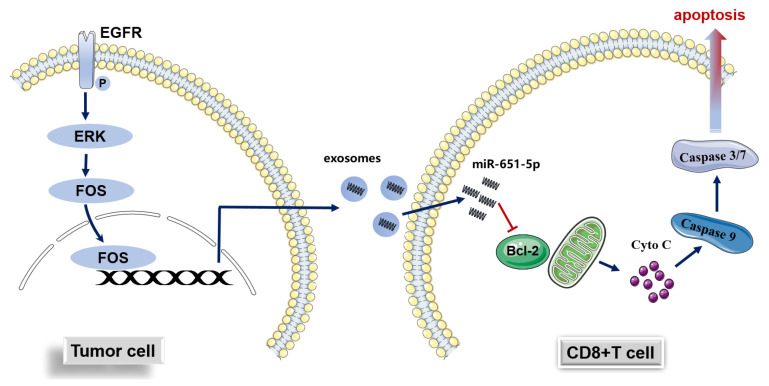
Hypothetical graph of this study. The expression of miR-651-5p is regulated by the EGFR signaling pathway, enters into CD8+T cells through exosomes, targets BCL2 to induce cell apoptosis, and finally induces immune escape.

Due to miR-651-5p promoting CD8+ T cell apoptosis, we explored whether the miR-651-5p antagomir could decrease T cell apoptosis, increase the infiltration number of CD8+ T cells, and further inhibit tumor progression. Firstly, we searched public databases to investigate whether miR-651-5p could influence the status of EGFR activation and found that it had no impact on key genes of the EGFR pathway (Supplementary Table S6), indicating that the antagomir probably had no impact on the EGFR pathway. We also detected whether it could influence the expression of AXL, HER2, and HER3 that are commonly involved in EGFR activation [22,23]. The results showed that it did not significantly influence their expression (Supplementary Figure S6). Subsequently, humanized mouse model was used to conduct the following research (Figure 6a). The miR-651-5p antagomir did not significantly inhibit tumor growth; however, the combination of the antagomir and PD-1 inhibitor significantly inhibited tumor growth (Figure 6b). The tumor scRNA sequencing showed that the antagomir increased mononuclear phagocytes (MPs) and T cell ratios (Table 1). To further analyze the T cell group, we found that the antagomir decreased the ratio of CD4+ Treg cells and CD8+ T exhaustion cells (Tex), while it increased the ratio of monocytes and helper T cells (Supplementary Tables S7 and S8). The antagomir improved the expression of the anti-apoptosis gene BCL2 in T cells, which was consistent with the above results, although there were no significant differences between different groups. It also promoted the expression of cell cytotoxicity-related gene perforin 1 (PRF1) (Figure 6c). IHC results showed that the infiltration of CD8+ T cells in the treatment groups had an increasing tendency, and immune cell apoptosis decreased in the treatment groups (Figure 6d,e). The above results suggest that miR-651-5p can promote the apoptosis of immune cells, and to inhibit the expression may enhance the anti-tumor efficacy of PD-1 inhibitors. The hypothetical of this study is shown in Figure 7.

## 4. Discussion

PD-1/PD-L1 inhibitors played significant roles in the era of immunotherapy. The inhibitor alone or combined with other therapies, such as chemotherapy and anti-angiogenesis drugs, prolonged the overall survival of *EGFR* wild type NSCLC patients. Nevertheless, *EGFR*-mutant NSCLC patients benefit little from the PD-1/PD-L1 inhibitor single drug compared with the wild type patients, which is an important question in clinical practice. Many studies focused on the issue; however, the mechanisms still need to be explored. In this study, we found that the ability of promoting CD8+ T cell apoptosis was stronger in *EGFR*-mutant cells than wild type cells. The pathway was through the EGFR/FOS/exosomal miR-651-5p/target cell BCL2 axis. The antagonist of miR-651-5p increased the ratio of TILs in the tumor, which could enhance the PD-1 antibody anti-tumor effect.

CD8+ T cells are crucial effector cells in anti-tumor immunotherapy. The infiltration number influences the immunotherapy effect [8], and apoptosis could decrease their number and activity [12,13,14,15]. In this study, we found that *EGFR*-mutant NSCLC cell lines had a stronger ability of promoting CD8+ T cell apoptosis than wild type cell lines. As CD4+ T cells mainly perform immune regulatory functions and CD8+ T cells mainly perform direct tumor-killing functions, it is efficient for tumors to combat CD8+ T cells stronger than other immune cells. The result is confirmed by other studies that tumors have a stronger inhibitory effect on CD8+ T cells than other immune cells [24,25,26,27]. These results indicated that the difference between the ability of promoting CD8+ T cell apoptosis is a potential mechanism of CD8+ T cell infiltration difference between *EGFR*-mutant NSCLC and wild type.

T cell apoptosis could be induced in different ways [28]. Our former study [11] and others [17] showed that tumor-derived exosomes could induce CD8+ T cell apoptosis. In this study, we confirmed the former results and also found that they could impair the tumor-killing effect of PBMCs. Further analysis suggested that the persistence of damage to the killing function of PBMCs by *EGFR*-mutant cell exosomes is stronger than that of wild type cell exosomes. This may be another mechanism that leads to a different efficacy of immunotherapy between *EGFR*-mutant and wild type NSCLC. Next, we explored which components of the exosomes promoted CD8+ T cell apoptosis and impaired the tumor-killing function. miRNAs are a wildly known component in exosomes, so small RNA sequencing was performed, and we found that miR-651-5p was rich in *EGFR*-mutant cell exosomes than wild type exosomes. miR-651-5p was reported to be associated with cancer growth/progression [29,30,31,32], virus infection [33], or used as disease biomarkers [34,35]; however, its role in the TME was not studied, as far as we know. In this study, we found that it could promote CD8+ T cell apoptosis; however, the influence of anti-tumor function was not significant, indicating there are other factors that paly the function.

Next, we examined the mechanisms of promoting apoptosis and being regulated. We found that it could target BCL2 and be regulated by the EGFR and FOS. FOS was reported to be regulated by the EGFR pathway [36,37]. From the above results, it could be speculated that *EGFR*-mutant NSCLC promotes CD8+ T cell apoptosis through EGFR/FOS/exosomal miR-651-5p/BCL2.

Given the role of miR-651-5p in promoting CD8+ T cell apoptosis, we explored whether the antagonist could decrease CD8+ T cell apoptosis and increase the infiltration number. Humanized mouse models are widely used for human immuno-oncology research. They are used to study the interactions between human tumors and the immune system to evaluate the efficacy of immunotherapies [38]. In this study, we used PC9 cell line-derived xenografts to perform the following research. It was found that the antagonist of miR-651-5p could increase the ratio, as well as the number of T cells. Further analysis showed that the ratio of helper T cells was increased, and the ratio of exhaustion CD8+ T cells was decreased. Helper T cells promote cytotoxic T lymphocytes (CTLs) to exert the anti-tumor functions or directly play the role themselves [39]. The increase in the cells should strengthen anti-tumor function. Limited by the cell number, we did not further classify subclasses of the cells. Exhaustion CD8+ T cells (CD8Tex) restricted tumor PD-1/PD-L1 inhibitor immunotherapy [40]. In this study, the miRNA antagomir improved the number and decreased the ratio of CD8Tex. The increased count of helper and CD8+ T cells may synergistically kill tumors. The expression of anti-apoptosis gene BCL2 was increased in the treatment group more than the control group, which was consistent with the previous results. The antagomir treatment group also increased the expression of some of the cytotoxicity genes in T cells, such as PRF1, indicating that miR-651-5p may activate the tumor-killing function; however, the effect may be weak, as the tumor volume in the group was not significantly decreased compared with the control group. Its main role was potentially on inducing T cell apoptosis. IHC staining confirmed that the CD8+ T cell infiltration increased, and immune cell apoptosis decreased in the treatment groups. The alteration of *BCL2*/*PRF1* gene expression, T cell infiltration and apoptosis were not statistically different, and the main reason might be due to the small number of the immune cells. However, the trend to some extent already proved the former results. To sum up, the mouse model results verified the cell line results and also indicated that decreasing T cell apoptosis was a potential way to strengthen immunotherapy effects.

The shortcomings in this study include but are not limited to the following aspects: Firstly, the immune cells treated in this study were from healthy volunteers rather than from the TME, and they were used to simulate, but could not replace, the immune cells in the TME. CD8+ T cells in the TME should be different from that in the PBMCs. We did not analyze which subtypes of CD8+ T cells were more sensitive to exosomes, such as the exhaustion T cells or the non-exhaustion cells. Without considering the economic situation, to obtain high-quality tumor samples and to combine multiple innovative technologies, such as scRNA-seq, spatial transcriptome and metabolome, will make the new findings easier, more convenient, and reliable than the traditional methods. Secondly, in the clinical practice of our center, the combinations of immunotherapy with other therapies were the main treatment methods, and the single PD-1/PD-L1 antibody treatment was used less frequently. So, the association between miR-651-5p expression and PD-1/PD-L1 inhibitor effect was not examined. Thirdly, due to the different components in *EGFR* mutation/wild type cell-derived exosomes, there may be different ways for exosomes to enter target cells [41]. The difficulty and tendency of entering immune cells may also be important factors for the two subtypes of tumors having different influences on the TME. We need further studies to investigate whether the different entry modes influence different functions of the immune cells. Anyway, given the role of exosomes in immune regulation, it is necessary to block the procedures of tumor-secreted exosomes entering target cells. In the future, the authors consider that there are at least three directions: 1. Is miR-651-5p a predictive factor for immunotherapy in NSCLC? 2. Which factor in the exosome inhibits the tumor-killing function of CD8+ T cells? 3. Do different immune cells have a tendency to absorb exosomes? What factors led to this situation? To resolve these issues will be of great help for our understanding of TMEs and immunotherapy in *EGFR*-mutant NSCLC.

## 5. Conclusions

In summary, *EGFR*-mutant NSCLC cells regulated miR-651-5p expression, which promoted T cell apoptosis through targeting BCL2. The miR-651-5p antagomir increased T cell infiltration and strengthened PD-1 antibody anti-tumor activity. Combining the miR-651-5p antagomir with the PD-1 antibody could be a potential treatment strategy in *EGFR*-mutant NSCLC immunotherapy.

## Figures and Tables

**Table 1:** The alteration of immune cells after treatment in the humanized mouse model.

Cell Type	Control	Antagomir	PD1 Antibody	Antagomir+PD1 Antibody
MPs	312 (3.68%)	388 (5.97%)	551 (9.16%)	721 (13.87%)
Cancer Cells	8026 (94.62%)	5858 (90.19%)	5287 (87.91%)	4338 (83.46%)
Mast Cells	80 (0.94%)	28 (0.43%)	62 (1.03%)	51 (0.98%)
T Cells	64 (0.75%)	221 (3.40%)	114 (1.90%)	88 (1.69%)

MPs, mononuclear phagocytes.

## Data Availability

The datasets used and/or analyzed in the current study were all included in this manuscript. If there is a further need, it can be provided upon reasonable request to the corresponding author.

## Supplementary Materials

The following supporting information can be downloaded at https://www.mdpi.com/article/10.3390/biomedicines13020482/s1, Figure S1: Exosomes were detected and uptook by PBMCs; Figure S2: Small RNA sequencing; Figure S3: miR-651-5p expression in cell lines, database and clinical samples; Figure S4: The potential TFs of miR-651-5p; Figure S5: The luciferase constructs in this study; Figure S6: The impact of miR-651-5p antagomir on EGFR relative receptors. Table S1: Main regents used in this study; Table S2: qRT-PCR primers; Table S3: The expression of miR-651-5p in different tumors and the normal tissues; Table S4: Apoptosis pathway analysis of miR-651-5p in ENCORI website; Table S5: The top five binding sites of miR-651-5p on FOS predicted in JASPAR website; Table S6: The potential binding sites of miR-651-5p on the key genes of EGFR pathway; Table S7: The subtypes of MPs in different groups; and Table S8: The subtypes of T cells in different groups.
