# Supplementary material for: EGFR-Mutant Lung Adenocarcinoma Cell-Derived Exosomal miR-651-5p Induces CD8+ T Cell Apoptosis via Downregulating BCL2 Expression
Source: Biomedicines. 2025 Feb 15;13(2):482. doi: 10.3390/biomedicines13020482 (PMC11852681; doi:10.3390/biomedicines13020482)
Supplement: Supplementary file 1 [file biomedicines-13-00482-s001.zip › supplementary figures.pdf]

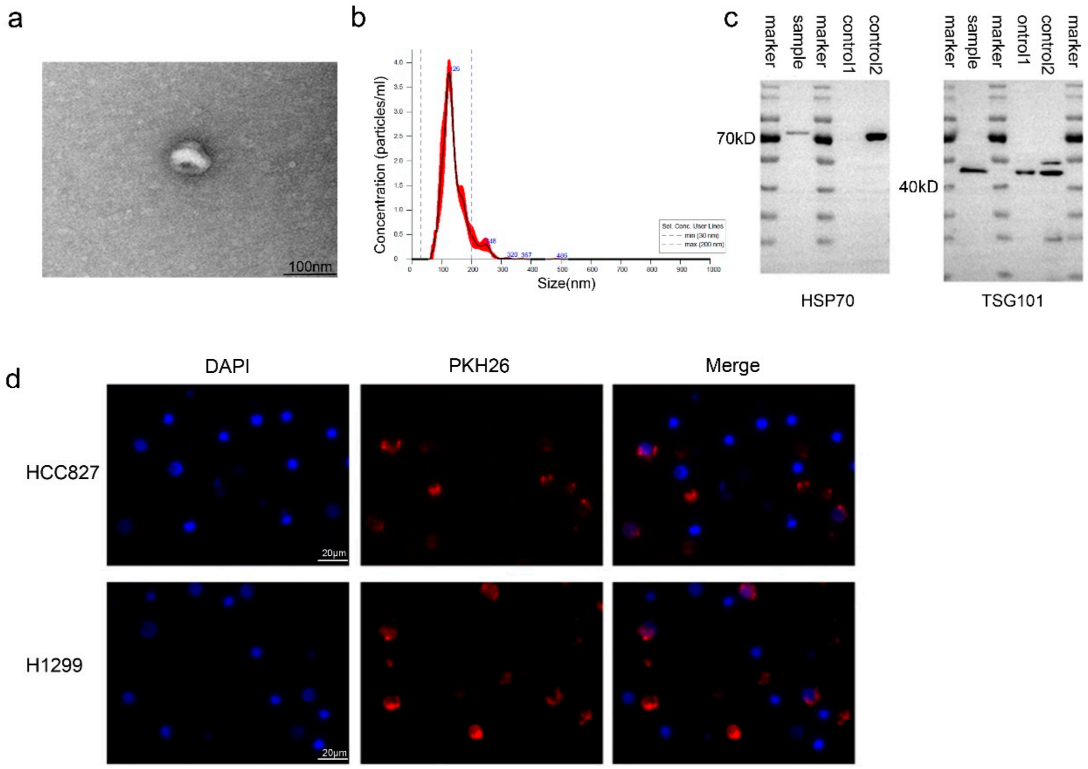

Supplementary figure S1: Exosomes were detected and uptaked by PBMCs. Exosomes were extracted and identified by transmission electron microscope (a), particle size (b) and western blot (c). Control1, supernatant of 293T cell culture medium; control2, 293T cell. (d) HCC827 and H1299 exosomes were labeled by red fluorescence dye PKH26, and used to treat PBMCs. The exosomes could be absorbed by PBMCs.

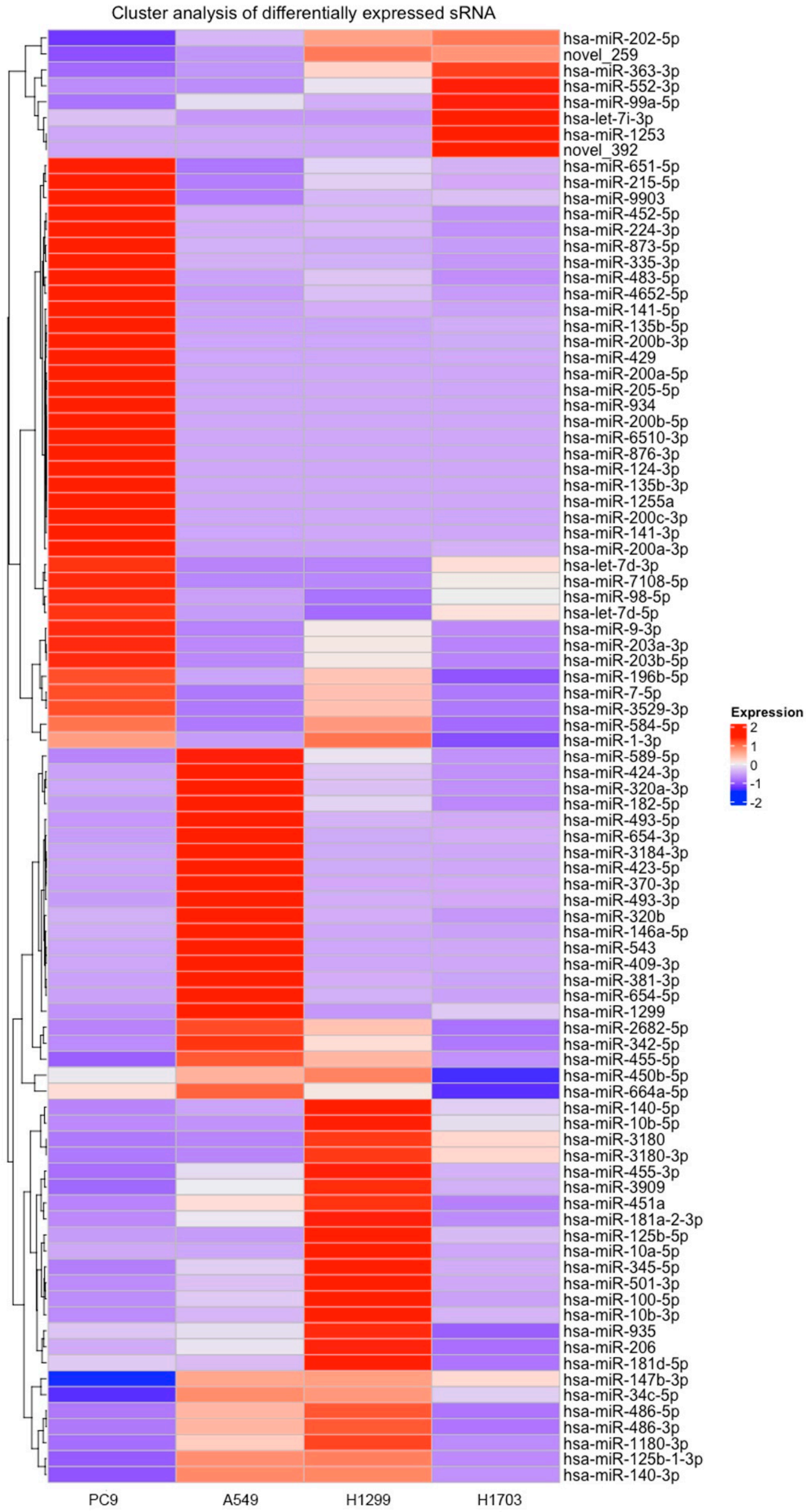

Supplementary figure 2: Small RNA sequencing. Small RNA sequencing detected the miRNA expression in cell secreted exosomes.

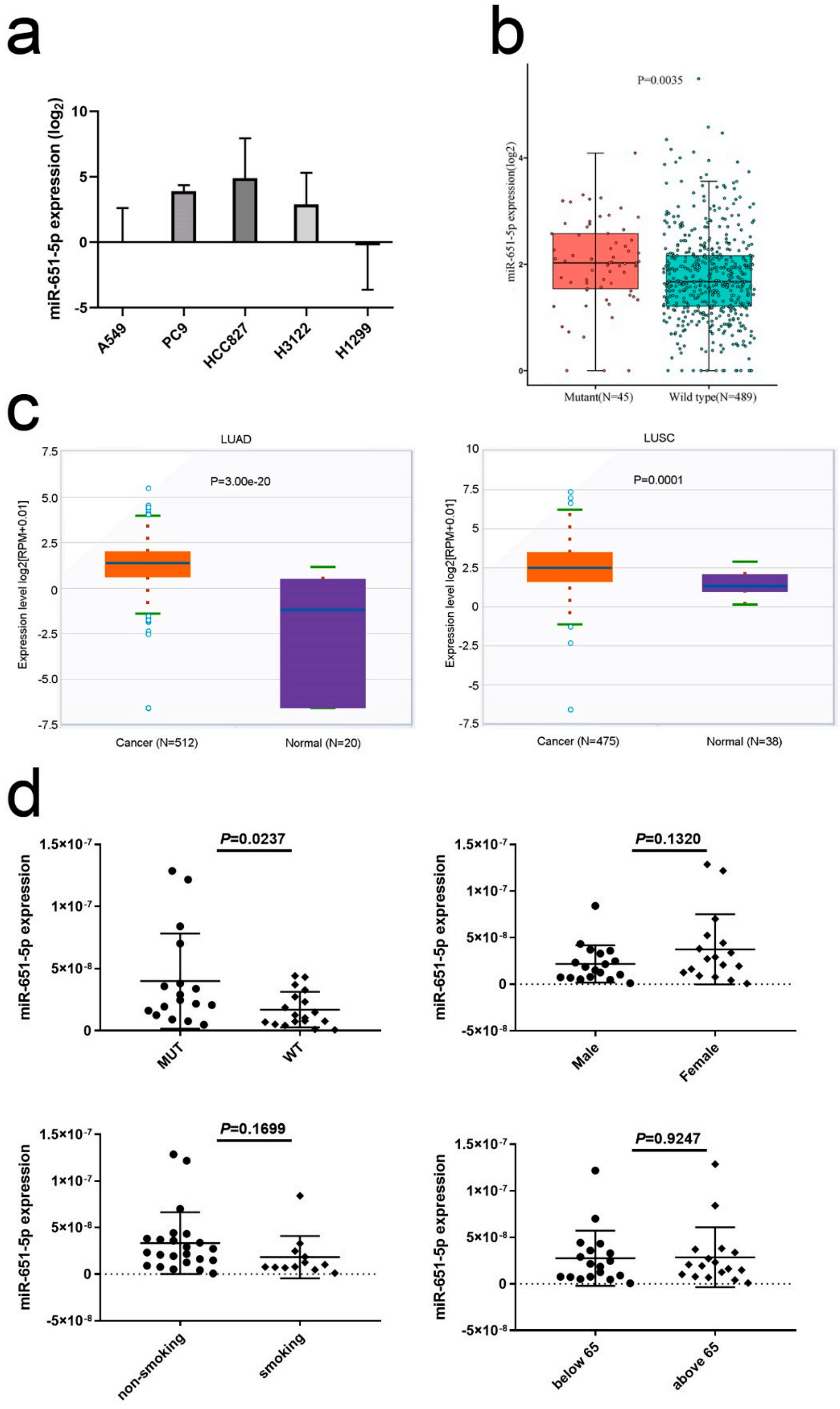

Supplementary figure 3: miR-651-5p expression in cell lines, database and clinical samples. (a) miR-651-5p expression was detected in cell lines by qRT-PCR. miRNAs expression was analyzed in TCGA database (b) and ENCORI database (c). (d) Thirty-five treatment naïve NSCLC samples were used to detect miR-651-5p expression, and it was significantly higher in the 17 *EGFR* mutant samples

than the 18 wild type samples. The expression was not significantly different between other clinical features.

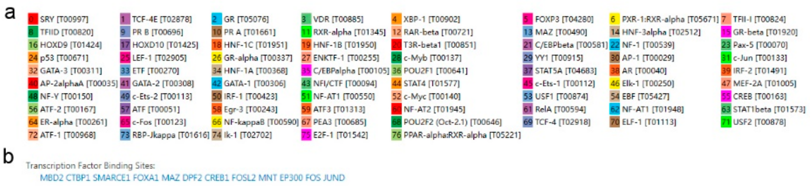

Supplementary figure 4: The potential TFs of miR-651-5p. PROMO (a) and Genecard (b) websites predicted that FOXA1/MAZ/CREB1/FOS were miR-651-5p potential TFs.

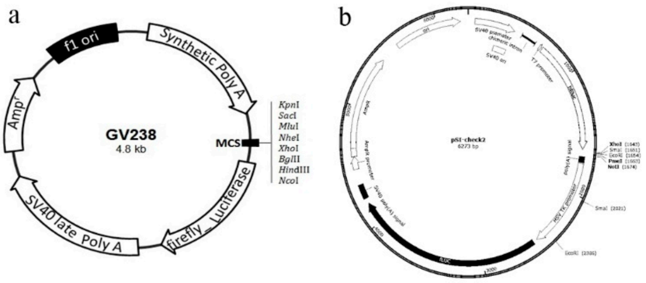

Supplementary figure 5: The luciferase constructs in the study. (a) The plasmid used to construct miR-651-5p upstream sequence. (b) The plasmid used to construct BCL2 3'UTR sequence.

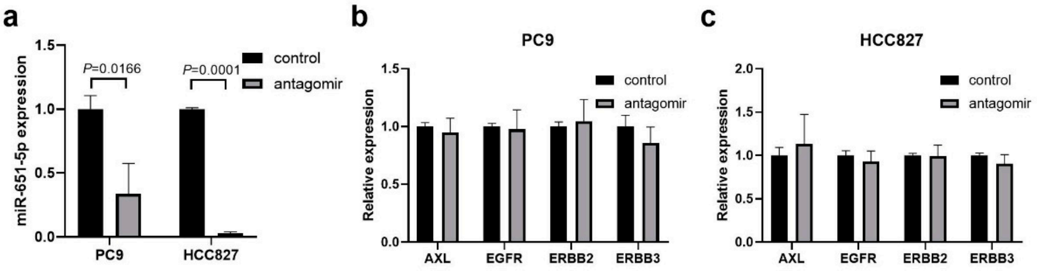

Supplementary figure 6: The impact of miR-651-5p antagomir on EGFR relative receptors. PC9 and HCC827 cell lines were treated by 100nM miR-651-5p antagomir for 48h, and the expression of miR-651-5p (a) as well as the mRNA expression levels of AXL, EGFR, ERBB2 and ERBB3 were detected by qRT-PCR (b and c).
