# Supplementary material for: EGFR-Mutant Lung Adenocarcinoma Cell-Derived Exosomal miR-651-5p Induces CD8+ T Cell Apoptosis via Downregulating BCL2 Expression
Source: Biomedicines. 2025 Feb 15;13(2):482. doi: 10.3390/biomedicines13020482 (PMC11852681; doi:10.3390/biomedicines13020482)
Supplement: Supplementary file 1 [file biomedicines-13-00482-s001.zip › supplementary tables.pdf]

Supplementary table 1: Main regents used in the study.

NO.	Name	Cat.No.	Brand	City	State	Country
1	Ficoll-Paque PREMIUM	17544203	GE Healthcare	Pittsburgh	Chicago	America
2	CD8 microbeads	130-045-201	Miltenyi Biotec	Bergisch Gladbach	/	Germany
3	fixable viability stain 620	564996	BD Pharmingen	Franklin Lake	New Jersey	America
4	Fc block	564220	BD Pharmingen	Franklin Lake	New Jersey	America
6	CD3	BV650	BD Pharmingen	Franklin Lake	New Jersey	America
7	Annexin-V	BUV395	BD Pharmingen	Franklin Lake	New Jersey	America
14	CD8	APC	BD Pharmingen	Franklin Lake	New Jersey	America
15	CD4	BV786	BD Pharmingen	Franklin Lake	New Jersey	America
17	cell counting kit-8	BS350B	Biosharp	Beijing	/	China
18	plasma exosome extration kit	EIQ3-02001	Wayen	Shanghai	/	China
19	miRNeasy Mini Kit	217004	Qiagen	Shanghai	/	Germany
20	Bulge-Loop miRNA qRT-PCR Starter Kit	C10211-2	Ribobio	Guangzhou	/	China
21	riboFECT CP Transfection Kit	C10511-05	Ribobio	Guangzhou	/	China
22	LipoFiter3.0	HB-LF3-1000	Hanbio	Shanghai	/	China
23	RNAiso Plus	9109	Takara	Dalian	/	China
24	RevertAid First Strand cDNA	K1622	Thermo Scientific	Waltham	Massachusetts	America
25	Dual-Luciferase® Reporter Assay System	E1910	Promega	Madison	Wisconsin	America
26	miR-651-5p antagomir		Ribobio	Guangzhou	/	China
27	pembrolizumab	A2005	Selleck	Texas	Houston	America
28	Single-cell sequencing	/	Singleron Biotechnologies	Nanjing	/	China
29	CD8 antibody	85336	Cell signaling	Boston	Massachusetts	America
30	Cleaved-caspase3 antibody	GB11532	Servicebio	Wuhan	/	China
31	Exosomes Tracer Kit	ESQ-R-001	Wayen	Shanghai	/	China
32	DAPI	G1012	Servicebio	Wuhan	/	China
33	E-PLATE 16	752724	Agilent	Santa Clara	California	America

Supplementary table 2: qRT-PCR primers.

Gene name	Forward primer (5'-3')	Reverse primer (5'-3')
MAZ	GCCCTTCAAATGTGAGAAATGT	ACCTTCATGTGGTCCGAAATAT
FOS	CTTCCCAGAAGAGATGTCTGTG	TGGGAACAGGAAGTCATCAAAG

FOXA1	GTTCTCCATCAACAACCTCATG	TATTGCAGTGCCTGTTCGTAT
CREB1	CTGATGGACAGCAGATCTTAGT	CTTCATTAGACGGACCTCTCTC
BCL2	GACTTCGCCGAGATGTCCAG	GAACTCAAAGAAGGCCACAATC
β-Actin	GAACGGTGAAGGTGACAGCAG	GTGGACTTGGGAGAGGACTGG
AXL	GCTGGAGGTGGCTTGGACTC	ACGGATGCTTGCGAGGTGAG
EGFR	TGTGCCACCTGTGCCATCC	ACCACCAGCAGCAAGAGGAG
ERBB2	AAGAGGTGACAGCAGAGGATGG	CCTGGATATTGGCACTGGTAACTG
ERBB3	GTGGTGAAGGACAATGGCAGAAG	TGAGGAGCACAGATGGTCTTGG

Supplementary table 3: The expression of miR-651-5p in different tumors and the normal tissues.

Cancer	Cancer full name	Cancer number	Normal number	Cancer expression	Normal expression	Fold change	p value	FDR
BLCA	Bladder Urothelial Carcinoma	408	19	4.7	0.87	5.43	4.50E-25	5.50E-23
BRCA	Breast Invasive Carcinoma	1085	104	2.97	1.45	2.05	4.20E-09	3.40E-08
CHOL	Cholangiocarcinoma	36	9	3.82	4.69	0.82	0.092	0.49
COAD	Colon Adenocarcinoma	450	8	3.69	0.27	13.69	3.80E-24	8.70E-23
ESCA	Esophageal Carcinoma	162	11	3.57	1.92	1.86	0.21	0.76
HNSC	Head and Neck Squamous Cell Carcinoma	497	44	4.52	3.06	1.47	0.06	0.18
KICH	Kidney Chromophobe	65	24	18.73	2.66	7.05	1.90E-08	3.00E-07

KIRC	Kidney Renal Clear Cell Carcinoma	517	71	2.13	1.13	1.89	0.00026	0.0014
KIRP	Kidney Renal Papillary Cell Carcinoma	289	32	3.44	1.73	1.98	6.10E-10	9.20E-09
LIHC	Liver Hepatocellular Carcinoma	370	50	4.33	4.48	0.97	0.1	0.3
LUAD	Lung Adenocarcinoma	512	20	3.37	0.69	4.85	3.00E-20	1.20E-18
LUSC	Lung Squamous Cell Carcinoma	475	38	8.77	2.94	2.99	0.0001	0.00069
PAAD	Pancreatic Adenocarcinoma	178	4	2.01	3.13	0.64	0.21	0.91
PRAD	Prostate Adenocarcinoma	495	52	2.3	1.01	2.28	2.30E-12	5.20E-11
STAD	Stomach Adenocarcinoma	372	32	3.1	1.36	2.28	2.40E-05	0.00025
THCA	Thyroid Carcinoma	509	58	4	4.97	0.8	0.012	0.042
UCEC	Uterine Corpus Endometrial Carcinoma	538	33	5	1.44	3.46	5.10E-07	4.10E-06

Supplementary table 4: Apoptosis pathway analysis of miR-651-5p in ENCORI website.

miRNA	log10(pval)	log10(FDR)	targetGeneNum	commonGeneNum
miR-651-5p	-5.92356	-5.02869	59407	87

Supplementary table 5: The top five binding sites of miR-651-5p on FOS predicted in JASPAR website.

Score	Relative score	Sequence ID	Start	End	Strand	Predicted sequence
12.6348	0.957450751	NC_000023.11:8124965-8127061	1153	1163	+	AATGATTCATT
11.0346	0.936107964	NC_000023.11:8124965-8127061	1153	1163	-	AATGAATCATT
9.80233	0.919673226	NC_000023.11:8124965-8127061	1539	1549	-	TCTTATTCATG
7.29996	0.886298897	NC_000023.11:8124965-8127061	1928	1938	+	AATTACTCAGC
6.40135	0.874314028	NC_000023.11:8124965-8127061	463	473	+	TGTGACTGATT

Supplementary table 6: The potential binding sites of miR-651-5p on the key genes of EGFR pathway.

Gene name	ENCORI website	TARGETSCAN website
PIK3CA	No	No
AKT1	No	No
RAF1	No	No
MAPK1	No	No
JAK1	Low binding score	No
STAT3	No	No

Supplementary table 7: The subtypes of MPs in different groups.

Cell type	Control	Antagomir	PD-1 inhibitor	Antagomir+PD-1 inhibitor
cDC2	11(4.72%)	31(8.42%)	14(2.81%)	29(4.48%)
Macrophages	153(65.67%)	189(51.36%)	235(47.19%)	328(50.70%)
Monocytes	57(24.46%)	134(36.41%)	230(46.18%)	269(41.58%)
Proliferating MPs	12(5.15%)	14(3.80%)	19(3.82%)	21(3.25%)

Supplementary table 8: The subtypes of T cells in different groups.

Cell type	Control	Antagomir	PD-1 inhibitor	Antagomir+PD-1 inhibitor
CD4Treg	10(25.64%)	28(17.72%)	15(18.29%)	8(14.29%)

CD4NaiveT	6(15.38%)	22(13.92%)	13(15.85%)	7(12.50%)
CD4Teff	5(12.82%)	16(10.13%)	7(8.54%)	10(17.86%)
CD4Tex	4(10.26%)	17(10.76%)	10(12.20%)	2(3.57%)
CD8Tex	3(7.69%)	7(4.43%)	14(17.07%)	3(5.36%)
HelperT	11(28.21%)	68(43.04%)	23(28.05%)	26(46.43%)
